# The Protective Effect of Lacidipine on Myocardial Remodeling Is Mediated by the Suppression in Expression of GPR78 and CHOP in Rats

**DOI:** 10.1155/2015/945076

**Published:** 2015-01-21

**Authors:** Yingxiao Ge, Gang Li, Baoxin Liu, Huixin Guo, Dongzhi Wang, Qiqiang Jie, Wenliang Che, Lei Hou, Yidong Wei

**Affiliations:** Department of Cardiology, Shanghai Tenth People's Hospital, Tongji University School of Medicine, Shanghai 200072, China

## Abstract

Lacidipine (LAC) is now widely used for the treatment of hypertension and further can prevent cardiac hypertrophy and remodeling. However, the underlying mechanism has not been fully understood. In this study, we examined the protective effects of LAC on cardiac remodeling in spontaneously hypertensive rats (SHR) and investigated the possible mechanism. Four weeks after administration of the designated drugs, blood pressure, left ventricular mass index (LVMI), and rterial pressure (MAP) were measured. The endoplasmic reticulum stress (ERS) parameters such as GRP78 and CHOP expressions in cardiomyocytes were also detected by immunohistochemistry. Results showed that the MAP in 0.36 and 0.72 mg/kg LAC groups was markedly lowered compared with that of the SHR control group (*P* < 0.01 or *P* < 0.05). Moreover, 0.72 mg/kg LAC could also significantly decrease the LVMI (*P* < 0.05). Simultaneously, the results of immunohistochemistry demonstrated that the expression of GRP78 and CHOP was significantly decreased by 0.72 mg/kg LAC (*P* < 0.05), respectively. Our present study suggested that LAC could lower blood pressure and could prevent left ventricular hypertrophy accompanied by inhibiting expression of GRP78 and CHOP in ERS.

## 1. Introduction

Hypertension, as a major public health problem, plays a significant role in a series of cardiovascular disorders. Moreover, it is generally considered that hypertension could increase the risk of cardiovascular events, such as cardiac failure, arrhythmia, and coronary heart disease. The pathogenesis of heart failure is myocardial hypertrophy and remodeling which results from the long-term effects of hypertension. However, the mechanism of how hypertension induces cardiac hypertrophy remodeling remains unclear. In recent years, accumulating evidences have suggested that endoplasmic reticulum (ER) dysfunction may be involved in these progressions [1, 2]. ER exists widely in eukaryotes and functions as essential manufactory for protein synthesis, folding, and secretion and the Ca^2+^ repository in myocytes [3]. Any adverse environmental change such as reactive oxygen species or calcium overload could affect the normal ER physiological function, which may result in the accumulation of incorrect folded proteins in ER lumen and finally induce ER stress (ERS). Several important ER chaperones such as GRP78 and C/EBP homologous protein (CHOP) are essential in proper protein folding and assembling [4, 5]. It has been accepted that the expression level of GRP78 could reflect the state of ERS and the ability of ER to restore homeostasis and CHOP could regulate several apoptotic signaling pathways leading to cell “suicide” which is triggered by excessive and perpetuated ERS [6, 7]. Calcium channel blockers are increasingly used for the treatment of hypertension. Lacidipine (LAC), a novel third-generation dihydropyridine calcium channel blocker, has been demonstrated to be effective for hypertension. Moreover, it could also prevent myocardial hypertrophy and remodeling [8, 9]. However, the underlying molecular mechanism remains unclear. The aim of this study was to examine the protective effects of LAC on myocardial remodeling in spontaneously hypertensive rats (SHR) and explore the possible mechanism.

## 2. Material and Methods

### 2.1. Animals and Reagents

Unless otherwise stated, all chemical reagents were purchased from Sigma Chemical Co. (St. Louis, MO, USA). Thirty adult male Wistar rats and fifty adult male SHR were purchased from Beijing Vital River Laboratory Animal Technology Co. Ltd. and housed in plastic cages with well-ventilated stainless steel grid tops at room temperature with a 12 h light/dark cycle. All of the experiments were performed in accordance with the Guidelines of Animal Experiments from the Committee of Medical Ethics at the National Health Department of China (Shanghai, China) and were approved by the Laboratory Center of Shanghai Tenth People's Hospital (Shanghai, China). Taking into account that SHR were generated from Wistar rats, 10 healthy Wistar rats were used as normal blank group to compare effects of lacidipine on SHR; 50 SHR were divided randomly into 5 groups: (i) SHR control group, (ii) captopril positive control group (captopril 35 mg/kg), (iii) LAC 0.36 mg/kg group, (iv) LAC 0.72 mg/kg group, and (v) LAC 1.44 mg/kg group. The rats were administered intragastrically with designated drugs daily in the following four weeks.

### 2.2. Measurement of Blood Pressure, Heart Rate, and Left Ventricular Mass Index (LVMI)

Blood pressure and heart rate were measured by tail manometry with RBP-1B-type rat tail cuff blood pressure meter (China-Japan Friendship Hospital Clinical Medical Research Institute, Beijing, China). After measurement, rats were anesthetized with pentobarbital sodium and the hearts were separated and rinsed several times with precooling phosphate buffered saline (PBS). The atria and right ventricular free walls were cut; left ventricular free walls and interventricular septum were weighted as left ventricles weight (LVW). Left ventricular mass index (LVMI) was calculated using the formula LVMI = LVW/BW (mg/kg).

### 2.3. Immunohistochemistry

Immunohistochemical analysis was performed to detect the expression of GPR78 and CHOP. Briefly, after consecutive steps including paraffin sectioning, dewaxing, antigen retrieval with citrate buffer, endogenous peroxidase deactivation with 3% H_2_O_2_, and antigen block with 5% BSA-PBS, sections were incubated overnight at 4°C with a rabbit polyclonal anti-rat GPR78 (Bjowofd, USA; dilution 1 : 250) or a rabbit polyclonal anti-rat CHOP (ACRIs, USA; dilution 1 : 250), followed by incubation with biotinylated horseradish peroxidase-conjugated anti-rabbit IgG secondary antibody (Abcam, USA) for 60 minutes at room temperature. Then, SABC solution (Streptavidin-Biotin Complex) was added and incubated for another 40 minutes. Subsequently, the sections were visualized with 3′3-diaminobenzidine solution (DAB kit) (Gene Tech) under an optical microscope.

### 2.4. Statistical Analysis

Data are expressed as mean ± SEM. Statistical analysis of data was performed by applying Student's *t*-test to determine the significance between two groups. Statistical significance of pairwise differences among three or more groups was determined using one-way analysis of variance (ANOVA) followed by post hoc test. *P* < 0.05 was considered statistically significant. Analysis was performed using SPSS for Windows (SPSS Inc., Version 17.0, Chicago, IL, USA).

## 3. Results

### 3.1. Effects of LAC on Heart Rate and Blood Pressure

Four weeks after the administration of designated drugs, the heart rate and MAP (*P* < 0.05 and *P* < 0.01, resp., Table 1) in SHR control group were both significantly increased when compared with normal blank group. However, captopril caused a sharp decrease in heart rate and MAP (*P* < 0.05 and *P* < 0.01, resp.) when compared with SHR control group. All LAC groups showed decrease in heart rate to some extent; moderate dose of LAC (0.72 mg/kg) could significantly cause bradycardia (*P* < 0.05), whereas both low and high dose of LAC showed no statistical significance (*P* > 0.05) compared with SHR control group. Similarly, the MAP was lowered after the treatments of different doses of LAC. The MAP was significantly reduced in 0.36 mg/kg LAC group (*P* < 0.05) and was the lowest in the 0.72 mg/kg LAC group (*P* < 0.01). However, the MAP in 1.44 mg/kg LAC group presented no statistical significance compared with SHR control group.

### 3.2. Effects of LAC on LVMI

After administration of aforementioned drugs for 4 weeks, there existed no significant difference in weight in all groups. Compared with blank group, heart weight in SHR control group increased significantly (*P* < 0.01, Table 2). Captopril positive control group and all LAC groups showed lowered heart weight. However, no significant difference was detected between LAC 0.36 mg/kg group and control group (*P* > 0.05). Similarly, both LAC 0.72 mg/kg and 1.44 mg/kg groups showed no statistical difference compared with SHR control group after taking body weight into account (*P* > 0.05).

### 3.3. Effects of LAC on GRP78 and CHOP Expression

**Figure 1:**
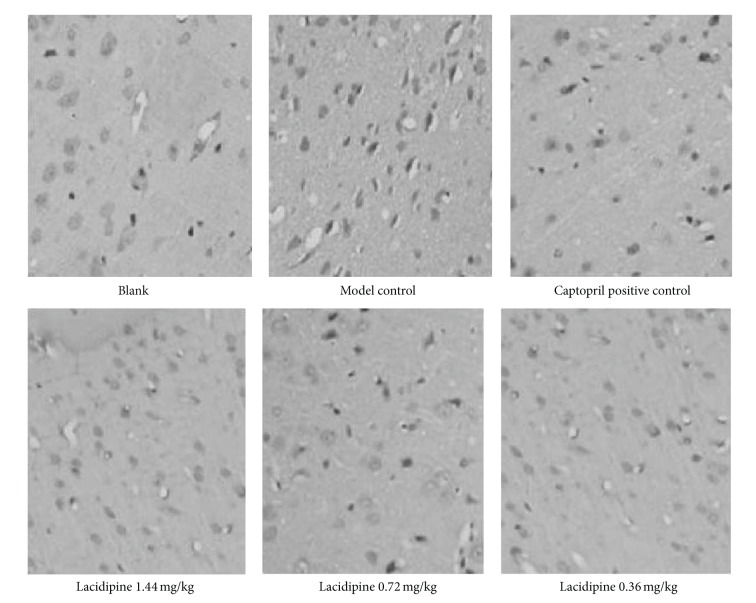
GRP78 expression level of different groups. Expression of GRP78 in myocardial tissue is detected by immunohistochemistry.

GRP78 is mostly generated in ERS to degenerate and clear the immature proteins; thus its expression could reflect the state of ERS and the ability of ER to restore homeostasis. As is shown in Figure 1, GRP78 expression showed a relevant lower level in normal blank group, whereas the expression of GRP78 demonstrated a significant increase in SHR control group. Both captopril and LAC inhibited GRP78 expression, but its expression in 0.36 mg/kg and 1.44 mg/kg LAC groups did not reach statistical significance compared with SHR control group (*P* > 0.05, Table 3).

**Figure 2:**
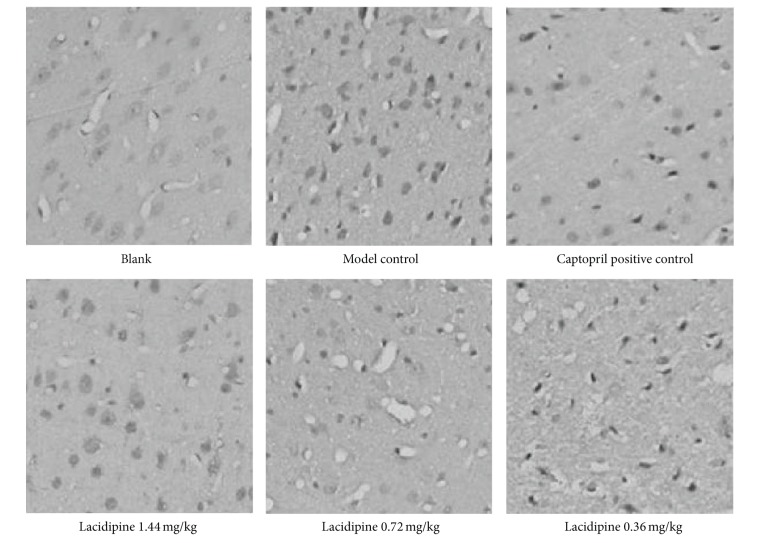
CHOP expression level of different groups. Expression of CHOP in myocardial tissue is detected by immunohistochemistry.

Similarly, CHOP expression was also in low level in normal blank group and increased significantly in SHR control group. The expression of CHOP decreased in captopril and LAC groups (Figure 2). Moreover, semiquantitative analysis showed that there were significant differences between captopril and LAC 0.72 mg/kg group when compared with SHR control group. However, CHOP expression presented no significant difference in 0.36 and 1.44 mg/kg LAC groups, respectively, when compared with SHR control group (*P* > 0.05, Table 3).

## 4. Discussion

Hypertension is a disease with high morbidity and hypertension-related cardiovascular diseases are the leading cause of mortality worldwide. However, the underlying molecular pathological mechanism of cardiac hypertrophy and remodeling induced by hypertension has not been fully understood. ERS is a sort of subcellular stress response which exerts a protective effect in clearance of impaired organelles and maintaining survival. Moreover, it is also involved in the pathogenesis of cardiovascular diseases including myocardial hypertrophy [10]; long-term and serious ERS may result in cell apoptosis and necrosis. GRP78 plays an important protective role in ERS and overexpression of GRP78 often indicates the disturbance of cell homeostasis [11]. CHOP, an apoptotic signaling molecular induced by persistent ERS [7, 12], acts as a common downstream regulation factor in different apoptotic signaling pathways. In normal physiological conditions, only low level CHOP expression was detected, but it increases significantly stimulation of calcium overload or accumulation of misfolded proteins. One histological characteristic of failing hearts is morphological development of ER [13]. Dally et al. had demonstrated in patients with heart failure that a marked increase of GRP78 expression was found [2], suggesting that ERS is associated with the pathophysiology of heart failure in humans. After transverse aortic constriction, similar changes were found in both hypertrophic and failing mice hearts and activation of CHOP was related to ER-initiated apoptosis [14].

In this study, we use SHR to observe the relationship between hypertension and heart remodeling and explore the possible mechanism underlying effects of LAC on hypertension. Our finding demonstrated that hypertension could result in increased heart weight, which is a symbol of cardiac hypertrophy and remodeling. Expression of GRP78 and CHOP was significantly increased in SHR. It is well known that calcium overload widely existed in hypertrophied myocytes, which could result in the upregulation of GPR78 to degenerate and clear these immature proteins. However, persistent hypertension will lead to excessive ERS and overexpression of CHOP which could finally result in apoptosis of myocytes. As a dihydropyridine calcium channel blocker, LAC could decrease blood pressure of SHR and inhibit the expression of GRP78 and CHOP with optimal dose of 0.72 mg/kg, which demonstrated that LAC could not only lower blood pressure but also protect left ventricular hypertrophy caused by pressure overload, improve cardiac function, and exert an antiapoptotic effect.

Limitations to our study should be noted. The study was performed using* in vivo* experimental systems. Additionally, the protection effects of LAC on cardiac hypertrophy and remodeling should be confirmed through* in vitro* experiments. Previous studies have demonstrated that Ca^2+^ inflow is associated with the expression of GRP78 and CHOP, whether the suppression of GRP78 and CHOP is mediated by LAC-induced blockade of Ca^2+^ inflow or antihypertensive function should also be determined [15, 16]. Moreover, studies are also required to elucidate the signaling pathways underlying this mechanism.

In conclusion, results of the present study showed that moderate dose of LAC could not only lower blood pressure but also exert a preventative effect in myocardial hypertrophy and remodeling to improve cardiac function. This protective effect may be associated with LAC-induced inhibition in the expression of GRP78 and CHOP.

## Figures and Tables

**Table 1:** Effects of lacidipine on heart rate and blood pressure.

Group	Heart rate (bpm)	MAP (mmHg)
Normal blank group	335.25 ± 26.2	112.57 ± 10.42
SHR control group	357.48 ± 29.5^*^	140.35 ± 11.73^**^
Captopril positive control group	342.48 ± 27.2^#^	127.12 ± 8.73^##^
Lacidipine 1.44 mg/kg group	351.18 ± 28.1	134.86 ± 9.55
Lacidipine 0.72 mg/kg group	341.32 ± 26.9^#^	128.65 ± 8.67^##^
Lacidipine 0.36 mg/kg group	346.48 ± 27.7	132.41 ± 9.23^#^

MAP: mean arterial pressure; ^*^ *P* < 0.05, ^**^ *P* < 0.01 compared with normal blank group; ^#^ *P* < 0.05, ^##^ *P* < 0.01 compared with SHR control group.

**Table 2:** Effects of lacidipine on left ventricular mass index.

Group	BW (g)	LVW (g)	LVMI (×10^−3^)
Normal blank group	305.1 ± 11.48	0.66 ± 0.08	2.15 ± 0.08
SHR control group	322.6 ± 9.86	1.06 ± 0.11^**^	3.26 ± 0.16^**^
Captopril positive control group	316.1 ± 10.86	0.92 ± 0.09^##^	2.91 ± 0.11^#^
Lacidipine 1.44 mg/kg group	320.5 ± 11.08	1.03 ± 0.10	3.19 ± 0.14
Lacidipine 0.72 mg/kg group	315.2 ± 10.18	0.89 ± 0.09^##^	2.84 ± 0.11^#^
Lacidipine 0.36 mg/kg group	317.8 ± 10.62	0.96 ± 0.10^#^	3.02 ± 0.12

BW: body weight, LVW: left ventricles weight, and LVMI: left ventricular mass index; ^*^ *P* < 0.05, ^**^ *P* < 0.01 compared with normal blank group; ^#^ *P* < 0.05, ^##^ *P* < 0.01 compared with SHR control group.

**Table 3:** Semiquantitative analysis of GRP78 and CHOP expression in myocardial tissue detected by immunohistochemistry.

Group	GRP78 (optical density)	CHOP (optical density)
Normal blank group	0.25 ± 0.02	0.08 ± 0.02
SHR control group	0.36 ± 0.04^*^	0.49 ± 0.07^*^
Captopril positive control group	0.29 ± 0.02^#^	0.25 ± 0.03^#^
Lacidipine 1.44 mg/kg group	0.31 ± 0.03	0.32 ± 0.04
Lacidipine 0.72 mg/kg group	0.30 ± 0.02^#^	0.27 ± 0.03^#^
Lacidipine 0.36 mg/kg group	0.33 ± 0.03	0.36 ± 0.04

^*^ *P* < 0.05 compared with normal blank group; ^#^ *P* < 0.05 compared with SHR control group.
